# Diffusion tensor imaging assessments to investigate motor impairment recovery after minor basal ganglia hemorrhage post-stereotactic surgery

**DOI:** 10.3389/fnins.2025.1526910

**Published:** 2025-05-09

**Authors:** Changpin Liao, Zhen Lu, Guiying Pan, Jing Ye, Shengde Nong, Rusli Bin Nordin, Jiancheng Liang, Muhammad Fattah Fazel, Nurul Hana Zainal Baharin

**Affiliations:** 1 Department of Neurosurgery, Baise People's Hospital, Baise, Guangxi, China; 2 Faculty of Medicine, MAHSA University, Kuala Langat, Selangor, Malaysia; 3 Department of Oncology Radiotherapy, Affiliated Hospital of Youjiang Medical University for Nationalities, Baise, Guangxi, China; 4 Department of Intensive Care Unit, Baise People's Hospital, Baise, Guangxi, China; 5 Faculty of Medicine, Bioscience and Nursing, MAHSA University, Kuala Lumpur, Malaysia; 6 School of Public Health, Youjiang Medical University of Nationalities, Baise, Guangxi, China; 7 Faculty of Pharmacy and Biomedical Sciences, MAHSA University, Kuala Lumpur, Malaysia

**Keywords:** basal ganglia hemorrhage, corticospinal tract, diffusion tensor imaging, post-stereotactic, surgery

## Abstract

**Objective:**

To investigate the efficacy of diffusion tensor imaging (DTI) in assessing the motor impairment resulting from minor basal ganglia hemorrhage post-stereotactic surgery.

**Methods:**

A total of 104 patients with minor basal ganglia hemorrhages (hematoma volume ≤ 15 ml) underwent DTI within 48 h and 1 month post-treatment. Patients were divided into two groups: 42 in the experimental group, receiving stereotactic surgery and medication, and 62 in the control group, receiving medication alone. The corticospinal tract (CST) of the posterior limb of the internal capsule was the region of interest (ROI) for assessing mean FA values on both sides. Fugl-Meyer motor function (FMF) scores were recorded within 48 h and 1 month post-treatment, and Modified Rankin Scale (MRS) scores at 6 months.

**Results:**

After 1 month of treatment, the FA values and FMF scores for the affected side of patients in the experimental group were 0.34 ± 0.17 and 67.84 ± 4.72, respectively, significantly surpassing those of the control group, which were 0.21 ± 0.06 and 45.38 ± 2.25 (*P* < 0.05). After 6 months of treatment, the experimental group exhibited MRS scores of 10 cases (23.81%) at grade 0, 12 cases (28.57%) at grade 1, 16 cases (38.10%) at grade 2, 2 cases (4.76%) at grade 3, 2 cases (4.76%) at grade 4, and 0 cases at grade 5. In contrast, the control group demonstrated 8 cases (12.90%) at grade 0, 12 cases (19.35%) at grade 1, 18 cases (29.03%) at grade 2, 15 cases (24.19%) at grade 3, 6 cases (9.68%) at grade 4, and 3 cases (4.84%) at grade 5. There are 18 cases (29.03%) in grade 2, 15 cases (24.19%) in grade 3, 6 cases (9.68%) in grade 4, and 3 cases (4.84%) in grade 5 within the control group. The experimental group showed a significantly better MRS score compared to the control group (*P* < 0.05).

**Conclusion:**

DTI can accurately evaluate the structural integrity of the CST in patients with minor basal ganglia hemorrhages following stereotactic surgery, particularly regarding the CST pathways involved in motor control, providing valuable guidance for clinical treatment.

## 1 Introduction

Basal ganglia hemorrhage (BGH) is the primary type of hypertensive intracerebral hemorrhage (HICH), accounting for roughly 40%−50% of all HICH instances (Lu et al., 2018). It generally causes impairment of the corticospinal tract (CST). The integrity of the CST is strongly associated with the patient's motor function, so more than 70% of BGH patients exhibit varied degrees of hemiplegia, which considerably affects their everyday activities (Lu et al., 2018). Treatment approaches for basal ganglia hemorrhage mainly encompass surgical intervention and pharmacological conservative management. The primary strategy is pharmacological conservative management for minor basal ganglia hemorrhages (hematoma volume ≤ 15 ml). Numerous studies (Yuan et al., 2022) have indicated that hypertensive cerebral hemorrhage patients with a hematoma volume over 15 ml can be expeditiously evacuated of hematoma by stereotactic surgery to enhance clinical outcomes. However, the clinical efficacy of this technique for basal ganglia bleeding with a hematoma volume of ≤ 15 ml remains contentious. No reports indicate which treatment technique is superior.

DTI is a crucial non-invasive method for evaluating the integrity of the CST and the patient's motor function recovery, serving as a significant reference for assessing neurological recovery and informing rehabilitation training (Schwarz et al., 2022; Puig et al., 2011). In this study, we utilized DTI technology to determine the extent of CST damage and changes in CST integrity before and after treatment in 104 patients with mild basal ganglia hemorrhage. Additionally, we evaluated motor function recovery and MRS scores to investigate the clinical efficacy of stereotactic surgery for minor basal ganglia hemorrhages.

## 2 Materials and methods

### 2.1 Study subjects

We conducted a retrospective analysis of the clinical records of 104 patients diagnosed with unilateral basal ganglia hemorrhage (hematoma volume ≤ 15 ml) who were admitted to the Neurosurgery Department of Baise People's Hospital from January 2021 to December 2021, adhering to specified inclusion and exclusion criteria. Among these 104 patients, 42 elected to undergo stereotaxic surgery, forming the experimental group, while the remaining 62 opted for conservative pharmacological treatment, constituting the control group. The patients' decisions were influenced by personal preference, the severity of their condition, and their comprehension of the treatment options. Given the subjective nature of patient selection, we ensured that differences in their conditions were considered at the time of selection. Retrospective data analysis was conducted to confirm that there were no significant differences in key clinical characteristics (e.g., age, and sex) between the two groups at baseline, ensuring their comparability.

Inclusion criteria: (1) adult patients aged over 18 with a history of hypertension; (2) unilateral basal ganglia hemorrhage verified by cranial CT within 24 hours of onset; (3) hematoma volume ≤ 15 ml as calculated by Tada's formula; (4) motor dysfunction in the limb contralateral to the lesion, with muscle strength ≤ 3; (5) MRS scores are all grade 3, indicating the need for assistance in daily activities; (6) absence of previous cerebral hemorrhage or neurosurgery; and (7) patients with comprehensive follow-up data.

Exclusion criteria: (1) individuals exhibiting unstable vital signs; (2) individuals with multiple organ failure, including cardiac, hepatic, or renal dysfunction; (3) individuals with significant cognitive impairment, poor adherence, and incapacity to engage in future rehabilitation training. All patients had DTI examination within 48 h of disease onset and again after 1 month of therapy. The Ethics Committee of Baise People's Hospital approved this study.

### 2.2 Methods

#### 2.2.1 Determination of hematoma volume

The patient underwent imaging with a Canon 320-row 640-slice high-end spiral CT (Aquilion 1 TSX-301C Canon Medical Systems Co., Ltd.) within 48 h of disease onset. The dimensions of the hematoma were measured individually, and the hematoma volume was computed using the Tada formula: hematoma volume (ml) = π/6 × length (cm) × width (cm) × height (cm).

#### 2.2.2 DTI Scanning and Image Processing

DTI scanning was performed using a 3T Siemens Skyra scanner with the following parameters: b values of 0 and 1,000, images in 20, gradient directions in 20, field of view (FOV) read 220, FOV phase 100, echo time (TE) of 85 ms, and repetition time (TR) of 3,700 ms. The original images underwent post-processing via the iMRI system, which enhanced the quality of the CST images through alignment of the original pictures, eddy-current correction, cephalic correction, and reconstruction of three-dimensional stereo images of the CST. Utilizing anatomical theory and FA maps, two ROIs were delineated on the 2D FA color maps of the DTI scans and were conducted using a 3T scanner (Skyra, Siemens, Germany) within 48 h of disease onset and 1 month post-treatment.

### 2.3 Therapeutic approach

Patients in the experimental group were required to complete the DTI examination before surgery, and the DICOM data from the DTI was integrated into the neurosurgical robot Remebot software system. A surgical approach was devised utilizing this data: the hematoma's center was designated as the target point, while the long axis of the hematoma served as the puncture pathway. This strategy circumvented critical functioning regions and blood arteries, with the puncture trajectory traversing the CST interstices. The patient was positioned supine, and under general anesthesia, the Mayfield head frame was applied for the surgery. Using a skull drill, create a vertical bone hole approximately 0.5 cm in diameter at the puncture site on the bone surface. The dura mater was incised in a cross pattern using a sharp scalpel, and bipolar electrocoagulation was employed to cauterize the dura mater and halt hemorrhage. A 12F soft silic1 drainage tube was inserted into the center of the hematoma along the puncture trajectory, and the hematoma was aspirated using negative pressure with a 5.0 ml syringe, which was halted upon encountering resistance or when 90% of the hematoma had been evacuated. The drainage tube was inserted through a subcutaneous tunnel measuring approximately 5.0 cm in length and securely anchored, following which the skin incision was closed in layers. A cranial CT scan was conducted within 24 H following the surgery. Urokinase was injected bi-daily into the hematoma cavity through the drain at 20,000 units per injection. The drain was occluded for 2 H after each infusion before being reopened. Cranial CT scans were performed every 2 to 3 days. The urokinase injections were terminated upon complete resolution of the hematoma, and the drain was extracted. Other treatments aligned with the control group. The control group received conservative treatment with medicine, which involved monitoring blood glucose and blood pressure, nourishing the nerves, preserving water-electrolyte balance, and aggressively preventing and addressing related problems.

### 2.4 Assessment metrics

The assessment metrics included gender, age, hemiplegic limb, onset blood pressure, onset time, hematoma volume, FA values, and FMF scores within 48 H of onset and 1 month post-therapy, as well as MRS scores after 6 months of treatment.

#### 2.4.1 Assessment of CST injury and recovery

**Figure 1:**
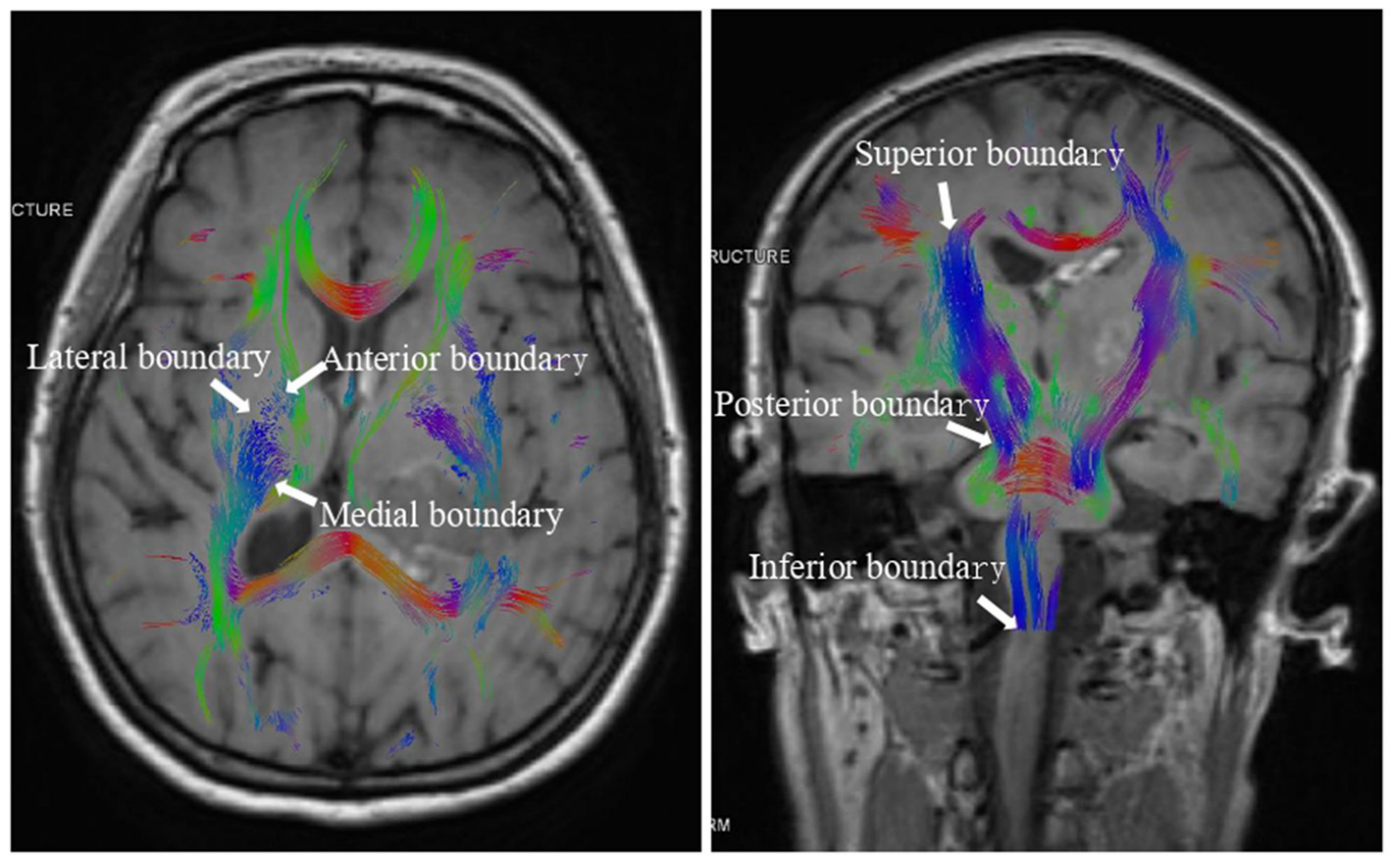
The boundary of the region of interest (ROI) of the posterior CST of the internal capsule.

On the CST 3D image, manually delineate the region of interest (ROI) of the posterior CST of the internal capsule, with the anterior boundary defined by the anterior limb of the internal capsule and the posterior boundary by end of the caudate nucleus, the posterior boundary by the midbrain, the medial boundary by the medial wall of the lateral ventricle, the lateral boundary by the putamen, the superior boundary by the cerebral cortex, and the inferior boundary by the medulla (Figure 1). Then, measure these regions' FA, MD, RD, and AD values. The DICOM data were imported into the Remebot software system to reconstruct the FA maps, directional coded color maps, and bilateral 3D CST maps to evaluate the extent of CST damage and recovery. FA, MD, RD, and AD values for the same patient were assessed three times by two senior neuroimaging physicians at Baise People's Hospital and the Affiliated Hospital of Youjiang Medical College of Nationalities. The mean of all measurements was utilized as the definitive value for this patient to ensure the reproducibility and generalizability of the results.

#### 2.4.2 Assessment of motor function and prognosis

The simplified FMF score was employed to evaluate motor function impairment in hemiplegic limbs within 48 h after disease onset, followed by a 1-month follow-up to document the recovery of motor function in the hemiplegic limbs. The FMF score ranges from 0 to 100, with a maximum score of 66 for upper limbs and 34 for lower limbs; a higher score indicates improved recovery of motor function in the hemiplegic limb. The clinical prognosis was assessed after 6 months of treatment using the Modified Rankin Scale (MRS), which ranges from grade 0 to 6. Grade 0: No symptoms, essentially normal living; Grade 1: Symptoms present but do not affect daily activities; Grade 2: Mild disability, but able to live independently; Grade 3: Moderate disability, requiring assistance with daily activities; Grade 4: Severe disability, requiring long-term care; Grade 5: Very severe disability, unable to care for oneself; Grade 6: Death. Two seasoned neurosurgeons evaluated all FMF scores and MRS scores independently, with a third senior neurosurgeon serving as an adjudicator in the event of a dispute.

### 2.5 Statistical analysis

Statistical analysis was performed using SPSS 20.0 software to compare general clinical data, MRS scores, FA values, and FMF scores between the experimental and control groups. Data are presented as (x ± s), with continuous variables analyzed using independent samples *t*-test and categorical variables analyzed using the chi-square test. A *P*-value of < 0.05 was considered statistically significant.

## 3 Results

### 3.1 Comparison of preoperative general clinical parameters across the two patient groups

No substantial difference existed between the two groups regarding gender, age, diastolic blood pressure upon onset, systolic blood pressure upon onset, time of disease onset at onset, FA, MD, RD, AD, and FMF scores for both the affected and healthy side within 48 h of onset (*P* > 0.05). Refer to Table 1 for further details.

**Table 1:** Comparison of preoperative general clinical parameters across the two patient groups.

**Contributing factors**		**Experimental group (*n =* 42)**	**Control group (*n =* 62)**	**t/χ^2^ value**	***P-*value**
Gender (*n*, %)	Male	23(54.76)	39(62.90)	0.689	0.423
	Female	19(45.24)	23(37.10)		
Age (*n*, years)	≥60	29(69.05)	34(54.84)	2.117	0.159
	< 60	13(30.95)	28(45.16)		
Hematoma volume ($\bar{\text{x}}\;\pm$ s, ml)		11.12 ± 3.77	9.98 ± 4.37	1.378	0.171
Systolic blood pressure on admission ($\bar{\text{x}}\;\pm$ s, mmHg)		159.32 ± 29.05	163.57 ± 25.61	0.786	0.434
Diastolic blood pressure on admission ($\bar{\text{x}}\;\pm$ s, mmHg)		103.74 ± 21.26	105.82 ± 18.39	0.531	0.597
Time from onset to admission ($\bar{\text{x}}\;\pm$ s, h)		10.25 ± 2.85	8.64 ± 5.77	1.674	0.097
The affected side within 48 h of onset ($\bar{\text{x}}\;\pm$ s)	FA	0.15 ± 0.11	0.17 ± 0.08	1.074	0.286
	MD(10^−3^mm^2^/s)	0.82 ± 0.14	0.79 ± 0.13	1.119	0.266
	RD(10^−3^mm^2^/s)	0.58 ± 0.15	0.61 ± 0.09	1.274	0.206
	AD(10^−3^mm^2^/s)	1.27 ± 0.11	1.23 ± 0.16	1.409	0.162
The healthy side within 48 h of onset ($\bar{\text{x}}\;\pm$ s)	FA	0.63 ± 0.12	0.64 ± 0.09	0.485	0.629
	MD(10^−3^mm^2^/s)	0.79 ± 0.18	0.83 ± 0.12	1.361	0.177
	RD(10^−3^mm^2^/s)	0.65 ± 0.14	0.62 ± 0.19	0.875	0.384
	AD(10^−3^mm^2^/s)	1.18 ± 0.22	1.21 ± 0.17	0.783	0.435
Within 48 h of onset ($\bar{\text{x}}\;\pm$ s)	FMF score of affected limb	25.27 ± 5.82	23.35 ± 7.16	1.444	0.152
	FMF score of the healthy limb	93.26 ± 3.68	92.74 ± 2.35	0.880	0.381

### 3.2 Comparison of clinical outcomes between the two groups of patients within 6 months of treatment

**Figure 2:**
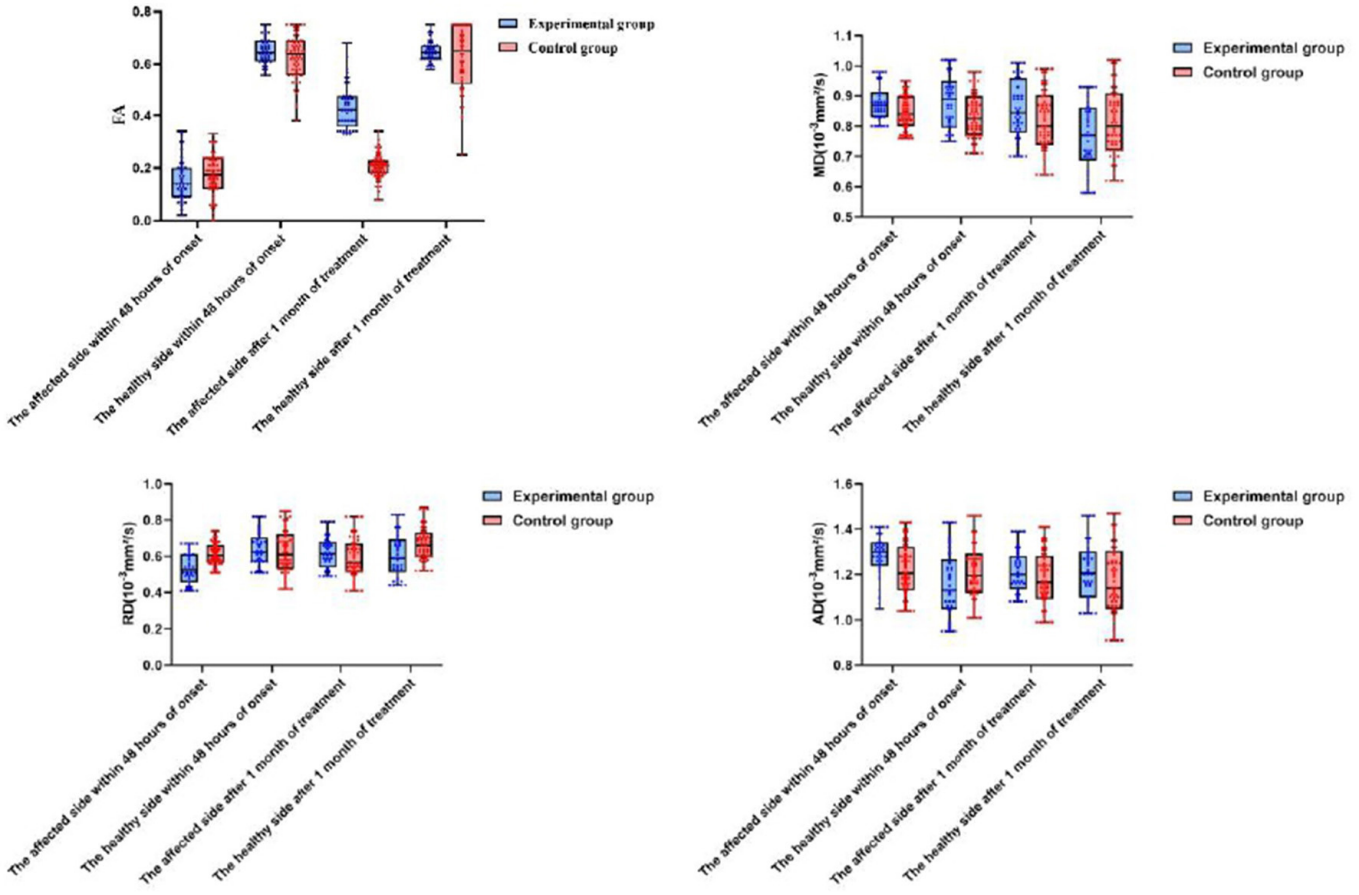
A comparison of changes in DTI within 48 h of onset and after o month of treatment between the two patient groups.

**Figure 3:**
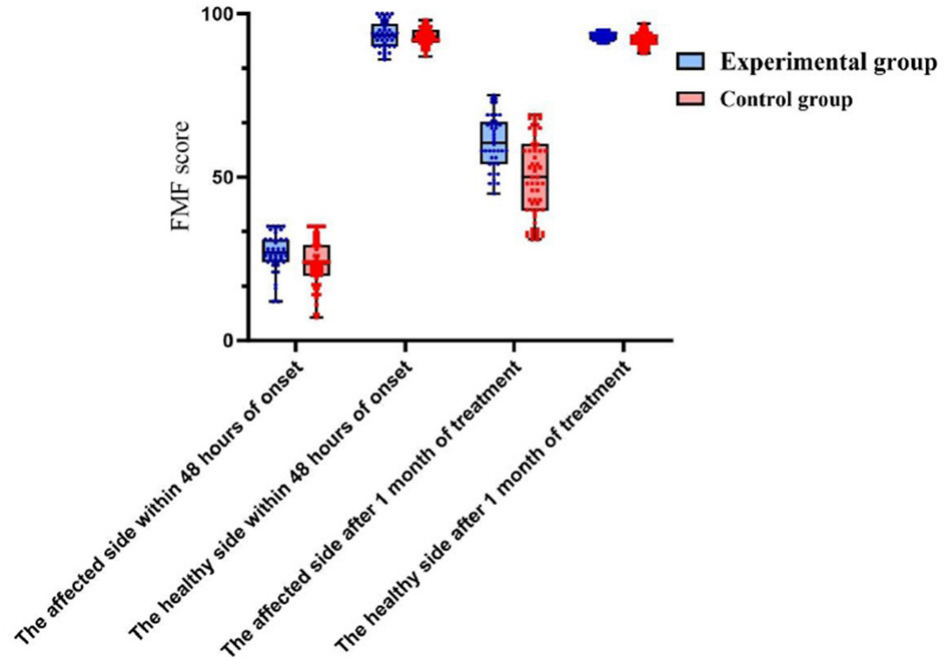
A comparison of FMF score between the two patient groups within 48 h of onset and 1 month after treatment.

Following 1 month of treatment, the FA value and FMF score for the afflicted side of patients in the experimental group were 0.34 ± 0.17 and 67.84 ± 4.72, respectively, demonstrating considerable improvement compared to the control group, which recorded values of 0.21 ± 0.06 and 45.38 ± 2.25 (*P* < 0.05). The FA value and FMF score of the healthy side in the experimental group were 0.65 ± 0.11 and 93.04 ± 1.89, respectively, which did not exhibit a statistically significant difference compared to the control group values of 0.62 ± 0.15 and 92.55 ± 2.33 (P>0.05). No significant differences in MD, RD, and AD were found between the affected and healthy sides in either group after one month of treatment (*P* > 0.05). After 6 months of treatment, the MRS scores in the experimental group comprised 10 cases (23.81%) of grade 0, 12 cases (28.57%) of grade 1, 16 cases (38.10%) of grade 2, 2 cases (4.76%) of grade 3, 2 cases (4.76%) of grade 4, and 0 cases (0%) of grade 5. In contrast, the control group exhibited 8 cases (12.90%) of grade 0, 12 cases (19.35%) of grade 1, and 18 cases (29.03%) of grade 2. In the control group, there were 18 cases (29.03%), 15 cases (24.19%) in grade 3, 6 cases (9.68%) in grade 4, and 3 cases (4.84%) in grade 5. No fatalities were reported in either group of patients. The MRS scores of patients in the experimental group were considerably superior to those of the control group after 6 months of treatment (*P* < 0.05). Refer to Table 2 and Figures 2, 3 for further details.

**Table 2:** Comparison of clinical outcomes between the two groups of patients within 6 months of treatment.

		**Experimental group (*n =* 42)**	**Control group (*n =* 62)**	**t/χ^2^ value**	***P*-value**
The affected side of 1 month after treatment ($\bar{\text{x}}\;\pm$ s)	FA	0.34 ± 0.17	0.21 ± 0.06	5.544	< 0.001
	MD(10^−3^mm^2^/s)	0.85 ± 0.13	0.82 ± 0.16	1.010	0.315
	RD(10^−3^mm^2^/s)	0.64 ± 0.11	0.59 ± 0.15	1.849	0.067
	AD(10^−3^mm^2^/s)	1.23 ± 0.12	1.19 ± 0.16	1.378	0.171
The healthy side of 1 month after treatment ($\bar{\text{x}}\;\pm$ s)	FA	0.65 ± 0.11	0.62 ± 0.15	1.109	0.270
	MD(10^−3^mm^2^/s)	0.75 ± 0.21	0.81 ± 0.19	1.514	0.133
	RD(10^−3^mm^2^/s)	0.63 ± 0.17	0.66 ± 0.13	1.019	0.311
	AD(10^−3^mm^2^/s)	1.24 ± 0.15	1.17 ± 0.24	1.680	0.096
1 month after treatment ($\bar{\text{x}}\;\pm$ s)	FMF score of affected limb	67.84 ± 4.72	45.38 ± 2.25	32.467	< 0.001
	FMF score of the healthy limb	93.04 ± 1.89	92.55 ± 2.33	1.133	0.260
MRS scores of 6 months after treatment (*n*, %)	Grade 0	10 (23.81)	8 (12.90)	11.874	0.037
	Grade 1	12 (28.57)	12 (19.35)		
	Grade 2	16 (38.10)	18 (29.03)		
	Grade 3	2 (4.76)	15 (24.19)		
	Grade 4	2 (4.76)	6 (9.68)		
	Grade 5	0	3 (4.84)		

### 3.3 Comparative analysis of CST charts within 48 h of onset and after 1 month of treatment between the two patient cohorts

**Figure 4:**
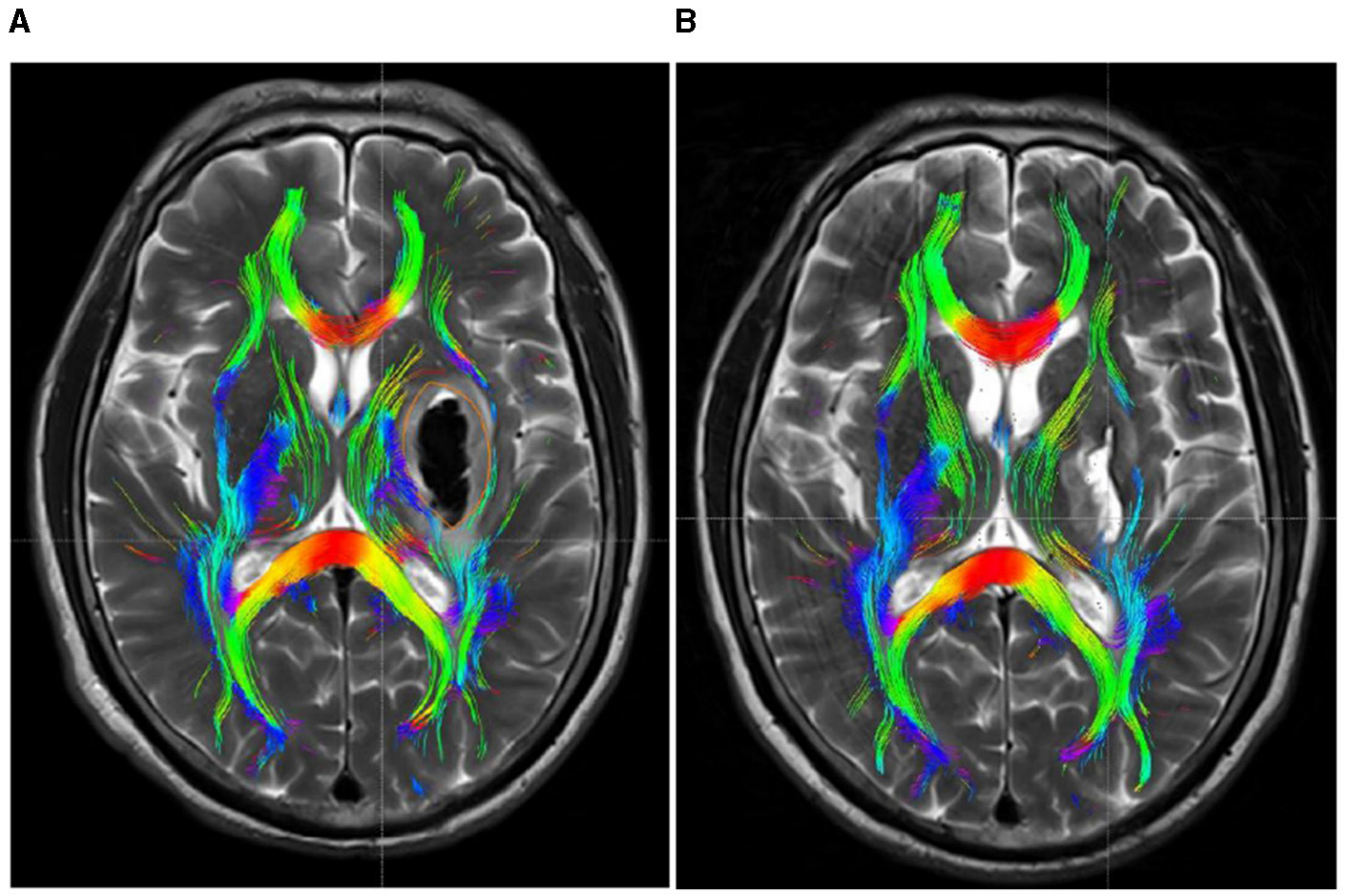
DTI picture of a patient exhibiting a minor basal ganglia hemorrhage, treated with stereotactic intervention within 48 h of admission **(A)** and 1 month **(B)**. A hematoma is located within the orange marker, resulting in distortion and deformation of the posterior limb of the internal capsule due to compression by the hematoma, which leads to disconnection of a portion of the CST. **(B)** Upon complete absorption of the hematoma, residual cavities emerge that do not exert pressure on the CST. The CST reverts to its natural anatomical position, with the initial damage primarily evident as additional repair connections.

**Figure 5:**
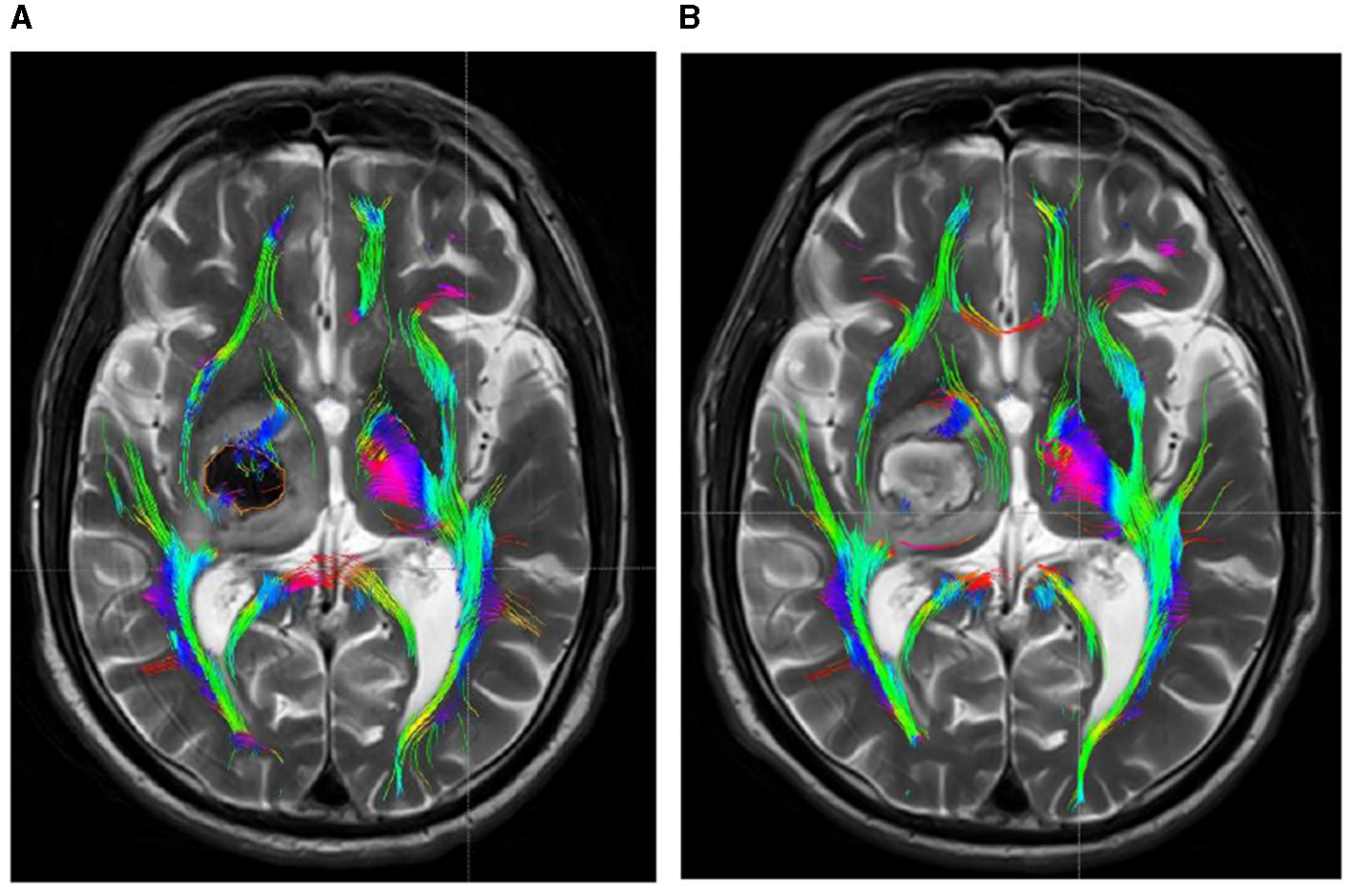
DTI picture of a patient with a minor basal ganglia hemorrhage, treated with conservative pharmacological treatment within 48 h of admission **(A)** and 1 month post-treatment **(B)**. **(A)** A hematoma is present within the orange marker, causing deformation and elevation of the posterior limb of the internal capsule due to compression by the hematoma. The CST has thinned due to partial rupture. Upon complete absorption of the hematoma, the posterior limb of the internal capsule remains marginally compressed due to cerebral edema, resulting in medial displacement of the CST. The original rupture exhibits minimal repair connections; however, the CST remains quite small.

Within 48 h post-onset, both patient groups exhibited persistent compression, displacement, and partial rupture of the corticospinal tract in the posterior limb of the internal capsule on the afflicted side. In addition, there was a significant reduction in the volume of the CST and a significant reduction in the number of fiber bundles. One month after treatment, the CST in the posterior limb of the damaged internal capsule exhibited restorative connections, with increased volume and fiber tracts. In the experimental group, the volume and fiber count of the affected CST were 1.45 ± 0.36 and 292.51 ± 13.96, respectively, while in the control group, these values were 1.03 ± 0.22 and 225.47 ± 16.81. The difference between the two groups was statistically significant (*P* < 0.05). Before and after treatment, there was no significant change in the CST within the unaffected side of the posterior limb of the internal capsule in either group. See Table 3 and Figures 4, 5 for details.

**Table 3:** Comparison of the volume and number of the CST between the two patient groups.

		**Experimental group (*n =* 42)**	**Control group (*n =* 62)**	**t/χ^2^ value**	***P*-value**
The healthy side within 48 h of onset ($\bar{\text{x}}\;\pm$ s, cm^3^)	The volume of the CST	2.72 ± 0.38	2.65 ± 0.42	0.866	0.388
	The number of the CST	375.19 ± 12.33	369.97 ± 21.64	1.414	0.160
The affected side within 48 h of onset (?x ± s, cm^3^)	The volume of the CST	0.74 ± 0.11	0.69 ± 0.23	1.310	0.193
	The number of the CST	216.52 ± 19.85	207.48 ± 27.36	1.838	0.069
The healthy side of 1 month after treatment ($\bar{\text{x}}\;\pm$ s, cm^3^)	The volume of the CST	2.79 ± 0.26	2.85 ± 0.17	1.424	0.158
	The number of the CST	364.88 ± 15.28	371.24 ± 17.72	1.896	0.061
The affected side of 1 month after treatment ($\bar{\text{x}}\;\pm$, cm^3^)	The volume of the CST	1.45 ± 0.36	1.03 ± 0.22	7.383	< 0.001
	The number of the CST	292.51 ± 13.96	225.47 ± 16.81	21.331	< 0.001

## 4 Discussion

The basal ganglia primarily consist of the nucleus accumbens, globus pallidus, caudate nucleus, and amygdala complex. Hemorrhage at these locations typically presents as contralateral limb hemiparesis, with a significant percentage of patients sustaining permanent paralysis (Zhang and Li, 2022; Arboix et al., 2007). CST injury of the posterior limb of the internal capsule is a significant factor contributing to contralateral limb hemiparesis in these patients. Basal ganglia hemorrhage induces corticospinal tract injury in the initial phases through localized ripping, compression, and displacement. Secondary damage resulting from subsequent brain tissue oedema, ischaemic necrosis, and chemicals from hematoma disintegration are significant contributors to CST damage. Consequently, comprehending the extent of CST injury, evaluating the hemiplegic condition of patients, and formulating specific rehabilitation programs would significantly contribute to restoring motor function and prognosis in individuals with basal ganglia hemorrhage.

The CST arises from the paracentral lobule and the precentral gyrus of the cerebral cortex, traversing the internal capsule, midbrain, pons, and spinal cord, constituting the brain's most considerable descending motor fiber. The posterior limb of the internal capsule, a component of the corticospinal tract, serves as the primary conduit for both efferent and afferent motor signals. Thus, a hemorrhage in the basal ganglia may lead to compromised transmission of motor signals. This syndrome blocks afferent and efferent motor signals in the contralateral limb, hence restricting random movements of the limbs. The corticospinal tract primarily innervates the extensor muscles of the ankle and fingers, which are crucial for sustaining dorsal and extensor functions, and it innervates upper limb functions to a significantly greater extent than lower limb functions (Staudt et al., 2000; Wolbrecht et al., 2018).

The secure and efficient excision of hematoma and mitigation of hematoma-induced damage to the CST are essential strategies for enhancing patient prognosis. Stereotactic surgery is currently recognized as 1 of the most effective minimally invasive procedures for patients with moderate cerebral hemorrhages with hematoma volumes over 15 ml (Yuan et al., 2022). Although this type of bleeding causes very modest damage to the corticospinal system, most individuals often leave behind varied degrees of contralateral limb hemiparesis, especially a few who may develop severe limb paralysis (Maeshima et al., 2002; Shaya et al., 2005). The primary treatment objective for these patients is to adequately recover motor function in hemiplegic limbs to enhance patient prognosis. The use of stereotactic surgery for minor basal ganglia hemorrhage has been contentious due to insufficient clinical efficacy and definitive imaging evidence.

DTI investigates the microstructure and functions of the CST primarily by applying anisotropy principles and analyzing water molecule diffusion in living tissues (Ghazi Sherbaf et al., 2019; Hampton et al., 2019). DTI technology, as a novel imaging and post-processing method, facilitates the anatomical localization of the CST and the evaluation of its integrity. The CST is classified into three grades according to its integrity (Cheng et al., 2015): grade 1 indicates the CST is intact surrounding the hematoma; grade 2 signifies partial interruption of the CST in certain regions; and grade 3 denotes complete disruption of the CST around the hematoma. Physicians can precisely assess a patient's limb motor function by reviewing the integrity of the corticospinal tract, as evidenced by prior research (Bürgel et al., 2006; Jang et al., 2006). The DTI parameters are radial diffusivity (RD), mean diffusivity (MD), FA, mode (MO), and axial diffusivity (AD). FA, the primary parameter indicating the extent of anisotropy in water molecule diffusion, represents the ratio of the anisotropy of water molecule dispersion to the total dispersion tensor. Its value ranges from 0 to 1, where 0 signifies maximal isotropic dispersion and 1 denotes maximal anisotropic dispersion. The peripheral layer of the CST comprises myelin tissue, and water molecules diffuse in the direction of CST propagation, exhibiting a significant disparity in diffusion intensity across each direction. When water molecules diffuse uniformly in all directions, it is termed isotropic; conversely, when the diffusion intensity varies, it is referred to as anisotropic. A greater degree of anisotropy correlates with an elevated FA value, indicating reduced damage to the CST, enhanced integrity, and improved signaling performance; conversely, a diminished FA value signifies increased damage to the CST, decreased integrity, and impaired signaling function (Qin et al., 2023). Although AD, RD, and MD values did not show significant differences in this study, they hold biological significance. These parameters are strongly linked to CST integrity, especially myelin degeneration and axonal injury. Therefore, even without statistical significance, they provide valuable insights into the mechanisms of CST injury and recovery. AD, RD, and MD changes may reflect myelin and axonal degeneration, which in turn affect FA values. Studies have shown that the magnitude of the FA value is positively correlated with the recovery of motor function in the contralateral limb (Jin et al., 2017). The integrity grading and FA value of CST derived from DTI technology can precisely evaluate CST damage and assess the regression of motor function in patients, thereby significantly guiding subsequent rehabilitation training and reducing disability rates (Zolkefley et al., 2021). This study primarily assessed the clinical efficacy of stereotactic surgery for minor basal ganglia hemorrhage by gathering FA values, FMF scores, and MRS ratings from two patient cohorts. Multiple clinical studies (Liu et al., 2024; Sondag et al., 2023) have shown that the prompt evacuation of hematomas from the brain through minimally invasive surgery can reduce their intracranial duration, thus mitigating brain injury caused by cerebral hemorrhage and significantly improving clinical outcomes. In this study, the FA value and FMF score after 1 month of treatment, as well as the MRS score after 6 months of treatment on the afflicted side in the experimental group, were considerably elevated compared to the control group.

Moreover, while the CST recovery of the hind limbs in the internal capsule of the experimental group was incomplete, the extent of improvement was markedly superior to that of the control group. Concurrently, DTI imaging revealed that the CST on the affected side of the internal capsule hind limb in the experimental group patients was predominantly or entirely reinstated to its normal anatomical position after 1 month of treatment, exhibiting no signs of CST compression, displacement, or deformation, thereby facilitating CST repair (Figure 1B). Despite the complete absorption of the intracerebral hematoma in the control group receiving conservative treatment within the same timeframe, the extended presence of the hematoma resulted in cerebral oedema in the affected region, causing compression of the corticospinal tracts, which subsequently experienced displacements, torsions, and deformations detrimental to the repair of the CST (Figure 2B). The DTI study results indicated that, after 1 month of therapy, the experimental group demonstrated markedly elevated FA values in the impacted CST and increased fiber bundle density relative to the control group. It suggests that the prompt removal of the hematoma mitigates its detrimental effects on the CST and promotes the CST's recovery. This study's results indicate that stereotactic surgery effectively enhances motor function and prognosis in patients; however, it is essential to acknowledge the potential risks and complications associated with the procedure, including postoperative hematoma enlargement, intracranial infections, and new-onset neurological deficits. These parameters are critical in evaluating the comprehensive clinical efficacy of the procedure. Yang et al. (2024) indicated that stereotactic surgery assisted by DTI imaging can effectively prevent injury to critical neural pathways and the CST, thereby facilitating the recovery of postoperative neurological function. In this study, individuals who underwent stereotactic surgery demonstrated markedly enhanced clinical efficacy after 6 months in comparison to those who got conservative pharmacological treatment. The results of this investigation align with these findings. This study involved procedures conducted by proficient surgeons utilizing high-precision stereotactic techniques aided by an advanced neurosurgery robotic system integrated with CTA and DTI image fusion technology. By circumventing blood vessels and perforating and draining through the CST interstice, collateral injury to blood vessels and the corticospinal tract was effectively averted during the surgical procedure. Furthermore, the aseptic technique was meticulously adhered to during the postoperative urokinase instillation into the hematoma cavity, and the drainage line was managed to prevent cerebral infection. Consequently, no novel brain deficits were detected in the experimental cohort. Concurrently, patients can achieve superior motor function recovery and a more favorable clinical prognosis, with its clinical efficacy markedly above that of conservative pharmacological treatment. Therefore, the authors concluded that early hematoma removal via stereotactic surgery in treating minor basal ganglia bleeding could establish advantageous conditions for the recovery of CST. Although the FA values, FMF scores, and MRS scores in the experimental group showed significant improvement during the 6-month treatment period, the lack of long-term follow-up prevented the assessment of the sustainability of these effects. Therefore, future studies should include long-term follow-up to assess the sustained impact of these improvements on patients' functional recovery and quality of life. Additionally, conducting a comprehensive evaluation of the potential risks and complications associated with the procedure is essential, while further refining research on the reliability and reproducibility of DTI assessments. These investigations will yield significant insights into the enduring advantages of stereotactic surgery for patients with small basal ganglia hemorrhage.

## 5 Study limitations

A limitation of this study is the potential bias in the manual delineation of the CST region (ROI). Although we have provided a detailed description of the methods used to generate the ROI, we acknowledge that the lack of clear anatomical markers defining the boundary between the posterior limb of the internal capsule and the adjacent CST may lead to variability in the manual delineation of the ROI by different evaluators. To reduce potential bias, we employed the method of “averaging multiple independent delineations.” Specifically, two senior neuroimaging physicians independently delineated the ROI of the same patient at Baise People's Hospital and Youjiang Ethnic Medicine College Affiliated Hospital, conducting three repeated evaluations of each delineation. The final result was the average of these evaluations. To ensure independence, each physician was blinded to the other's evaluation during the delineation process. Additionally, the limited follow-up period of 6 months restricted our ability to assess long-term clinical prognosis. Extending the follow-up to 1 year to evaluate FA, FMF, and MRS scores could have strengthened the study's conclusions.

## 6 Conclusion

Our findings demonstrate that DTI imaging technology can assist clinicians in assessing the extent of damage and recovery of the CST in the affected basal ganglia region of patients with small basal ganglia hemorrhage. This technology plays a critical role in evaluating the improvement of motor dysfunction and prognosis following stereotactic surgery, underscoring its substantial potential for clinical application.

## Data Availability

The original contributions presented in the study are included in the article/supplementary material, further inquiries can be directed to the corresponding authors.
