# Correction: Diffusion tensor imaging assessments to investigate motor impairment recovery after minor basal ganglia hemorrhage post-stereotactic surgery

**DOI:** 10.3389/fnins.2025.1729005

**Published:** 2025-11-10

**Authors:** Changpin Liao, Zhen Lu, Guiying Pan, Jing Ye, Shengde Nong, Rusli Bin Nordin, Jiancheng Liang, Muhammad Fattah Fazel, Nurul Hana Zainal Baharin

**Affiliations:** 1 Department of Neurosurgery, Baise People's Hospital, Baise, Guangxi, China; 2 Faculty of Medicine, MAHSA University, Kuala Langat, Selangor, Malaysia; 3 Department of Oncology Radiotherapy, Affiliated Hospital of Youjiang Medical University for Nationalities, Baise, Guangxi, China; 4 Department of Intensive Care Unit, Baise People's Hospital, Baise, Guangxi, China; 5 Faculty of Medicine, Bioscience and Nursing, MAHSA University, Kuala Lumpur, Malaysia; 6 School of Public Health, Youjiang Medical University of Nationalities, Baise, Guangxi, China; 7 Faculty of Pharmacy and Biomedical Sciences, MAHSA University, Kuala Lumpur, Malaysia

**Keywords:** basal ganglia hemorrhage, corticospinal tract, diffusion tensor imaging, post-stereotactic, surgery

Author Rusli Bin Nordin was erroneously spelled as Rusli Nordin.

Affiliation “Faculty of Medicine, MAHSA University, Kuala Langat, Selangor, Malaysia” was omitted for author Changpin Liao. This affiliation has now been added for author Changpin Liao.

The original version of this article has been updated.

